# First Detection of West Nile Virus by Nasopharyngeal Swab, Followed by Phylogenetic Analysis

**DOI:** 10.3390/pathogens13111023

**Published:** 2024-11-20

**Authors:** Carlo Zuddas, Sergio Piras, Stefano Cappai, Federica Loi, Giulia Murgia, Giantonella Puggioni, Giovanni Savini, Federica Monaco, Andrea Polci, Fabrizia Valleriani, Giorgia Amatori, Valentina Curini, Maurilia Marcacci, Germano Orrù, Antonio Ledda, Elena Poma, Riccardo Cappai, Ferdinando Coghe

**Affiliations:** 1 Istituto Zooprofilattico Sperimentale della Sardegna, 07100 Sassari, Italy; giulia.murgia@izs-sardegna.it (G.M.); giantonella.puggioni@izs-sardegna.it (G.P.); 2 Laboratorio Analisi Chimico Cliniche e Microbiologia, Presidio Ospedaliero Duilio Casula, Azienda Ospedaliero Universitaria di Cagliari, Monserrato, 09042 Cagliari, Italy; sepiras92@gmail.com (S.P.); e.poma@aoucagliari.it (E.P.); r.cappai@aoucagliari.it (R.C.); fcoghe@aoucagliari.it (F.C.); 3 Osservatorio Epidemiologico Regionale della Sardegna, Istituto Zooprofilattico Sperimentale della Sardegna, 09125 Cagliari, Italy; stefano.cappai@izs-sardegna.it (S.C.); federica.loi@izs-sardegna.it (F.L.); 4 WOAH Reference Laboratory for WND, Istituto Zooprofilattico Sperimentale dell’Abruzzo e del Molise, Campo Boario, 64100 Teramo, Italy; g.savini@izs.it (G.S.); f.monaco@izs.it (F.M.); a.polci@izs.it (A.P.); f.valleriani@izs.it (F.V.); g.amatori@izs.it (G.A.); v.curini@izs.it (V.C.); m.marcacci@izs.it (M.M.); 5 Dipartimento di Scienze Chirurgiche, Università degli Studi di Cagliari, 09125 Cagliari, Italy; orru@unica.it; 6 Dipartimento di Ematologia/CTMO, Ospedale A. Businco, 09121 Cagliari, Italy; leddantonio@tiscali.it

**Keywords:** arbovirus, encephalitis, flavivirus, nasopharyngeal swab, next-generation sequencing (NGS), West Nile virus

## Abstract

West Nile Virus, an arthropod-borne RNA virus, may result in severe neurological disease. West Nile neuroinvasive disease is characterized by meningitis, encephalitis, and possible acute flaccid paralysis. Here, we report a case of neuroinvasive WNV in a 65-year-old woman hospitalized for hyperpyrexia, chills, intense asthenia, and continuous vomiting. Within days, her clinical condition worsened with the onset of severe neurological symptoms, leading to her death within 10 days despite supportive therapies being administered. The diagnosis of West Nile disease was made through nucleic acid amplification testing (NAAT) on blood and cerebrospinal fluid. However, in the final stages of the illness, cerebrospinal fluid collection was not possible due to the patient’s critical condition, and a nasopharyngeal swab was used instead. The nasopharyngeal swab facilitated the collection of a sample, which was subsequently analyzed for the presence of the virus and allowed for sequencing, showing that it was a strain that had been circulating in Sardinia for some time and had demonstrated its pathogenicity by causing the death of a hawk in 2021. This case report highlights the rapid progression and severity of WNV infection, particularly in vulnerable individuals, and suggests the potential utility of nasopharyngeal swabs as a less invasive option for sample collection. It also underscores the potential for the zoonotic transmission of the virus from birds to humans through vectors, emphasizing the importance of monitoring and controlling WNV outbreaks, especially in regions where such circulation is observed.

## 1. Introduction

West Nile Virus (WNV) is a vector-borne pathogen that has gained global attention due to its extensive geographic distribution and potential to cause severe illnesses in humans. WNV was first identified in the West Nile district of Uganda during surveillance for the yellow fever virus in 1937 [1].

WNV is an enveloped, positive-sense RNA virus belonging to the *Orthoflavivirus nilense*, a species that has recently undergone a nomenclature update within the genus *Orthoflavivirus*, family *Flaviviridae*. It is comprised in the Japanese encephalitis (JE) virus serocomplex together with JE virus, St. Louis encephalitis (SLE) virus, and Murray Valley encephalitis virus [2].

A large variety of WNV strains have been detected worldwide. Based on their genetic differences, they have been divided into eight lineages. Of these, lineages 1 and 2 are by far the most widely distributed [3]. They have been reported in many areas, including America, Africa, Europe, and Australia, and they are responsible for major human and equine outbreaks, particularly in Europe [4]. In Italy, the first evidence of virus circulation was reported in 1998 in horses in the region of Tuscany. Following this first occurrence, a national program for monitoring the virus’s presence and circulation in Italy has been implemented. Over the years, however, the importance of interaction between different professional figures and integration among various surveillance systems such as veterinary, entomological, and human became evident. Since 2016, veterinary surveillance (animal and entomological), essential for risk estimation, and human case surveillance have been integrated into a single plan, which has been included in the 2020–2025 Surveillance and Response Plan for Arboviruses (PNA) [5].

The WNV enzootic transmission cycle involves birds and mosquitoes, with wild birds acting as reservoir hosts. Up to 45 species and 8 genera [6] of mosquitoes with different host-feeding preferences can transmit WNV; however, species of the genus *Culex* are considered to be the main vectors [7]. Humans, horses, and other vertebrates susceptible to WNV infection are accidental or dead-end hosts [8] due to their inability to develop a level of viremia sufficient to infect a new vector and to complete the transmission cycle [9].

WNV infection in humans can result in various clinical pictures. Most (80%) infected individuals remain asymptomatic, but a few (20%) may experience West Nile Fever (WNF), which is characterized by mild to moderate flu-like manifestations. In a very low percentage (1%) of individuals, however, WNV infection may cause neuroinvasive West Nile disease (WNND), which can clinically manifest as acute flaccid paralysis, meningitis, encephalitis, or ocular disorders, representing the most severe and potentially fatal complications, especially among elderly individuals or those with compromised immune systems [10].

The diagnosis of acute WNV infection in humans is based on the detection of WNV RNA in clinical specimens by nucleic acid amplification methods (NAAT) or demonstrating a specific immune response against the virus.

Regarding molecular diagnosis, it has to be said that the real-time polymerase chain reaction (qRT-PCR) assay on serum, plasma, and cerebrospinal fluid (CSF) samples has limited efficacy for detecting WNV infection owing to the low viral titers and transient viremia WNV causes in humans. In the urine, the presence of WNV RNA can last longer. Due to the challenges associated with WNV viral detection, serological testing for specific antibodies currently constitutes a valid diagnostic approach. However, the prolonged presence of anti-WNV IgM antibodies in serum for up to 3 months after infection and the cross-reactivity among different flaviviruses make it difficult to diagnose an acute WNV infection based solely on these tests. Additionally, the low viral load and brief duration of viremia (typically 2–6 days) pose difficulties for nucleic acid amplification testing (NAAT) of blood and organ donors [11]. Thus, in light of these limitations and to avoid the complications and hurdles associated with lumbar puncture, improved and/or additional protocols and methods for WNV diagnosis and screening are needed.

## 2. Case Presentation

On 18 September 2022, a 65-year-old woman began experiencing high fever and chills. On 21 September, she presented herself at the emergency room of a peripheral hospital in the Sardinian healthcare network. A chest x-ray was performed with negative results. The woman had a previous history of diffuse large B cell lymphoma diagnosed in July 2022, a form of non-Hodgkin lymphoma, with metastatic locations in the central nervous system, for which they underwent four cycles of R-CHOP (rituximab, cyclophosphamide, hydroxydaunorubicin, vincristine, and prednisone). On 22 September, she was admitted to a center specializing in oncohematology in the regional hospital network; the patient appeared in a lucid and oriented state, although they slightly slowed down. This was followed by intense asthenia and continuous emesis in association with hyperpyrexia. Following the increase in fever, a blood culture test, a routine biochemical and hematological examination, was performed and an initial empirical therapy was administered for suspected infection. The day after hospitalization, on 23 September, when she woke up, she was found to have global aphasia; the patient was awake with open eyes but uncooperative, intermittently engaging with the examiner and unable to answer simple questions. Furthermore, she was found to have right-sided hemiplegia. Her vital parameters, such as saturation, blood pressure, and heart rate, were good. Urgent neurological counseling was requested and routine biochemical and hematological examination, brain scanning, and a lumbar puncture were performed. The results of the cerebrospinal fluid (CSF) examination were as follows: 61 mg/dL glucose (normal range: 50–75 mg/dL), 88.1 mg/dL protein (normal range: 15–45 mg/dL), and 122 mEq/L Cl (normal range: 115–130 mEq/L). In the serum sample, the glucose concentration was 111 mg/dL (normal range: 70–100 mg/dL) and the total protein concentration was 6.4 g/dL (normal range 6.6–8.3 g/dL). The results of the brain scan and the CSF film array for bacteria and viruses were negative. On 24 September, the patient’s general condition remained stable. However, symptoms such as aphasia, fever, high heart rate, and her ability to open her eyes in response to verbal stimuli persisted. As a result, healthcare professionals adapted the appropriate therapy to the new clinical situation.

Blood cultures for aerobic and anaerobic bacteria were negative throughout the period. Upon questioning the family members, it emerged that, before hospitalization, the patient stayed in an environment heavily burdened by the presence of mosquitoes. As a result, a blood sample was collected from the patient and sent to the laboratory for a NAAT test for WNV. The decision to investigate WNV was based on a combination of clinical, anamnesis, and epidemiological factors. The patient’s clinical presentation, including fever, neurological symptoms, and systemic manifestations, was consistent with the typical profile of WNV infection. Moreover, the patient’s medical history lacked any risk factors commonly associated with dengue, Zika, or other arboviruses, such as recent travel to endemic areas or exposure to known vectors of these viruses. Furthermore, the Sardinia region, where the patient resided, is endemic to WNV, with recent reports of virus circulation in humans and animals. Other arboviruses, such as dengue, chikungunya, and Zika, were not considered primary suspects due to the absence of these viruses in local surveillance reports during the relevant period. The test result confirmed that it was a form induced by WNV and, since there is no specific therapy against this pathogen, the doctors limited themselves to continuing the supportive therapy as already decided at the time. On 25 September, the patient’s general condition worsened considerably; she was unresponsive to stimuli and was hemiplegic on the right side, but she responded to localized pain on the left side and opened her eyes in reaction to painful stimuli. The doctors asked for a specialist infectious disease consultation, which confirmed that the patient presented a clinical picture of encephalitis. Concurrently, routine laboratory tests were conducted, and a serum sample was collected for serological analysis of the West Nile Virus. Additionally, a nasopharyngeal swab was taken to carry out molecular investigations on this biological matrix for the detection of WNV, although the protocols did not provide for it; a sample of cerebrospinal fluid was not collected due to the impossibility of performing another lumbar puncture, as it was considered highly risky given the patient’s condition. The laboratory investigations for a possible diagnosis were carried out at the Regional Reference Laboratory for Arboviruses located in the “Duilio Casula” Hospital of the University Hospital of Cagliari. WNV RNA was detected in both matrices examined: serum and nasopharyngeal swab. As there is no specific therapy for WNV, the medical therapy remained unchanged.

On 27 September, the patient’s clinical condition worsened further: she was hemodynamically stable, eupneic, on oxygen therapy, but not responsive to verbal, tactile, or pain stimuli and had flaccid paralysis of the upper limbs; her lower limbs were in spastic flexion. Her heart, chest, and abdominal scans were negative. On 29 September, the patient died. As indicated in the National Surveillance Plan, positive samples were sent to the National Reference Laboratory for West Nile Disease (WND) and Usutu as designated by the Italian Ministry of Health at the Istituto Zooprofilattico Sperimentale dell’Abruzzo and Molise “G. Caporale” in Teramo to confirm the test results. Here, a confirmatory real-time PCR was performed and, subsequently, the viral genome was sequenced.

## 3. Materials and Methods

In this clinical case, serological and virological methods were used to detect the eventual presence of WNV antibodies and RNA from serum samples and nasopharyngeal swabs.

For detecting viral genetic material, a real-time RT-PCR using the cobas^®^ 4800 system from Roche Diagnostics (Basel, Switzerland) was performed at the AOU laboratory in Cagliari. The cobas^®^ 4800 system consists of the cobas^®^ x 480 instrument for sample preparation and purification, and the cobas^®^ z 480 analyzer for real-time RT-PCR. The kit used was LightMix^®^ Modular West Nile Virus, produced by Roche Diagnostics (Basel, Switzerland).

An ELISA (enzyme-linked immunosorbent assay) was used to detect WNV IgG and IgM specific antibodies. The test performed at the AOU laboratory in Cagliari utilized the Chorus TRIO instrument (DIESSE Diagnostica Senese, Monteriggioni, Italy), an automatic multiparametric random access system for individual immunometric assays with ready-to-use single-test devices. The kits used were Chorus West Nile Virus IgM e Chorus West Nile Virus IgG, produced by DIESSE Diagnostica Senese (Monteriggioni, Italy).

### Next-Generation Sequencing (NGS) and Phylogenetic Analysis

Nucleic acids were extracted from the nasopharyngeal swab sample and preserved in UTM^®^ (Universal Transport Medium) using the MagMAX CORE Nucleic Acid Purification Kit (Applied Biosystems, Thermo Fisher Scientific, Life Technologies Corporation, Austin, TX, USA). Amplification reactions were conducted with specific one-step quantitative reverse-transcription polymerase chain reactions (qRT-PCRs) to detect WNV-L1 and -L2 [12] and all known lineages of WNV, following the established protocols [13].

Whole-genome sequencing (WGS) was performed starting from RNA purified using a specific WNV-L2 amplicon-based protocol [14]. Specifically, the reverse-transcription (RT) step was performed using Random Hexamers (50 μM) (Thermo Fisher Scientific, Waltham, MA, USA), 10 mM dNTPs mix (Promega, Madison, WI, USA), RNaseOUT™ (Thermo Fisher Scientific, Waltham, MA, USA), and SuperScript™ IV Reverse Transcriptase (Thermo Fisher Scientific, Waltham, MA, USA). The RT mix was incubated at 23 °C for 10 min, 50 °C for 10 min, and 80 °C for 10 min. Subsequently, two different amplification mixes were prepared using 2.5 μL of cDNA and 2.7 μL of 10 μM primer pool 1 and 2 designed ad hoc for WNV-L2 (Supplementary Materials) and Q5^®^ High-Fidelity DNA Polymerase (New England Biolabs, Ipswich, MA, USA) [14] with the following thermal cycling protocol: 98 °C for 30 s, 35 cycles of 95 °C for 15 s and 65 °C for 5 min, and final cooling step at 4 °C. The PCR products were combined and then purified using the Qiaquick PCR Purification kit (Qiagen, Leipzig, Germany). DNA quantification was performed using the Qubit DNA HS Assay Kit on the Qubit fluorometer (Thermo Fisher Scientific, Waltham, MA, USA).

Subsequently, library preparation was carried out by Illumina DNA prep kit (Illumina Inc., San Diego, CA, USA) and sequenced on the NextSeq500 using NextSeq 500/550 Mid Output Reagent Cartridge v2 (300 cycles) with standard 150 bp paired-end reads (Illumina Inc., San Diego, CA, USA). Quality control and trimming of the raw reads were performed using FastP v0.23.1 [15] and FastQC tool v0.11.5 (Bioinformatics Group, Brabraham Institute, Cambridge, UK). Mapping analysis was performed by iVar v1.3.1 [16] and using West Nile Nea Santa-Greece-2010 as the reference (HQ537483.1).

Phylogenetic analysis was conducted on a dataset of 198 WNV-L2 reference sequences representative of different geographic regions retrieved from the NCBI database (Supplementary Materials). The first strain of WNV-L2 identified in Europe in 2004 was used as the outgroup (DQ116961.1).

All sequences were aligned using Unipro UGENE v.45.1 [17] with the MAFFT algorithms [18], and only coding regions (CDS) were considered for subsequent phylogenetic analyses. Maximum likelihood phylogenetic trees were obtained using IQTREE v.1.6.12 [19] with the following command lines: “iqtree-s MafftAlign_WNV-L2_CDS outgroup.fa-st DNA-m TEST-bb 10000-alrt 10000-abayes”.

## 4. Results

Neither IgM nor IgG antibodies were detected in the serum sample (IgM index value 0.1; IgG index value 0.1; index values: absent, <0.9; borderline, 0.9–1.1; present, >1.1). According to the guidelines for the definitive diagnosis of WNVD, the case was considered certainly positive because virus RNA was found in the two biological matrices examined.

The sample tested positive for WNV-L2 with a Ct value of 37 while testing negative for WNV -L1. Illumina sequencing produced 1,210,584 raw reads. Following trimming, 206,790 reads remained with an average length of 145 nucleotides (nt). Mapping analysis produced a consensus sequence of 10,305 nt in length with a vertical coverage of 573X and a horizontal coverage of 82%.

**Figure 1:**
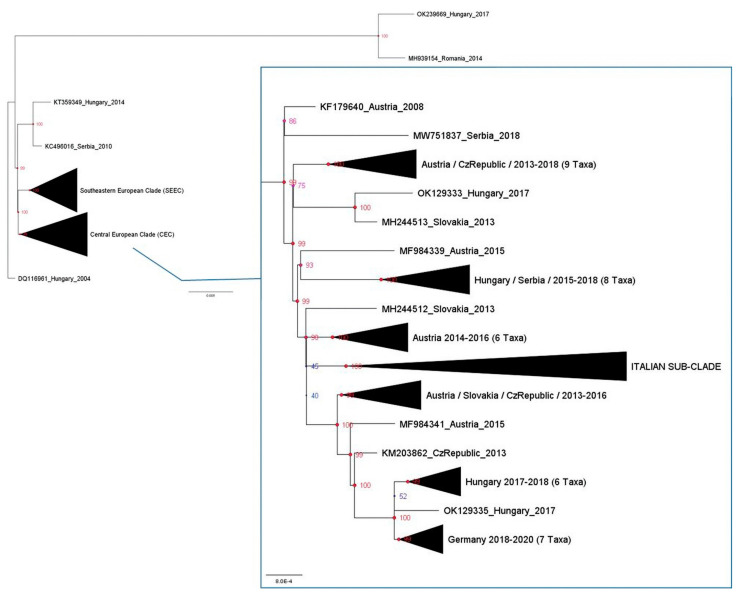
The evolutionary history was inferred using IQTREE 1.6.12 using command line: iqtree –s AlignWNVL2_CDS.fa –st DNA –m TEST –bb 10,000 –alrt 10,000 –abayes. The optimal tree is shown. The percentage of posterior probabilities in which the associated taxa clustered together are shown next to the branches. The tree is drawn to scale, with branch lengths measured in substitutions per site and optimized by maximum likelihood on original alignment. Model selection was carried out on all datasets using ModelFinder implemented in IQTREE. The rate variation among sites was modeled with GTR + F + I + G4, and the best fit model was chosen according to Bayesian Information Criterion (BIC). The tree is radicated to DQ116961 Hungary 2004 sequence. The node shapes are sized by posterior probabilities (PPs). The red nodes have posterior probabilities > 80%, while the blue nodes have posterior probabilities < 80%.

Phylogenetic analysis revealed the presence of two primary clades: the Central Europe clade (CEC) and the Southeastern European clade (SEEC) (Figure 1). The novel WNV-L2 strain NRG6464 (GenBank acc. no. PP625328.1) is placed in the CEC which contains the vast majority of the Italian WNV-L2 sequences, including other Sardinian WNV-L2 sequences dating back to 2012.

**Figure 2:**
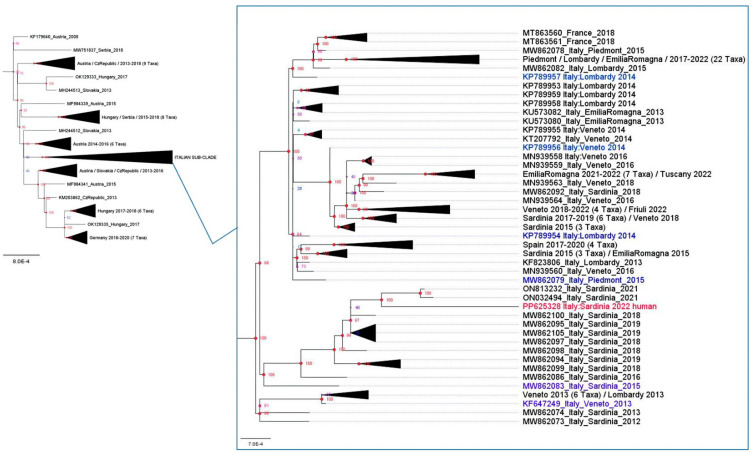
Enlargement of the Italian sub-clade of the tree reported in Figure 1. This clade includes all the Italian WNV-L2 from 2012 to 2022. The WNV-L2 strain NRG6464 (PP625328) is colored in red.

Within the CEC, the Italian sub-clade comprises three clusters, one of which includes only Sardinian sequences from 2015 to 2022 (Figure 2).

The WNV-L2 strain NRG6464 is placed here and shares a high degree of similarity (99.72%) with the WNV strain detected in a goshawk (*Accipiter gentilis*) from the Sassari province (ON813232.1), which showed neurological signs before succumbing to the infection (Figure 2). The implication drawn from this similarity is significant. This suggests that the strain of WNV responsible for the goshawk’s death is pathogenic for both birds and humans. Furthermore, this indicates that this strain likely originated from the internal circulation of the virus within Sardinia.

## 5. Discussion

WNV stands out as a pathogen that well adapts to the evolving landscapes shaped by globalization and climate change. Initially considered a virus of minor significance capable only of causing sporadic and self-limiting outbreaks, WNV has undergone a significant transformation over the years. Thanks to its remarkable adaptability, the virus has spread globally, becoming the most widely distributed arbovirus in the world [20]. This transformation has elevated WNV to an increasingly serious threat to public health, with outbreaks primarily affecting birds, humans, and horses [8]. The increasing spread of its geographical range and its capacity to trigger severe outbreaks underscore the critical need for continuous surveillance and effective control measures to tackle this escalating public health issue.

The first significant European epidemic of WND occurred in Romania in 1996. This epidemic gained attention as it marked the first occurrence of WND predominantly impacting an urban setting, leading to a high number of symptomatic human cases with involvement of the central nervous system [21].

In Italy, the first documented outbreak of WND occurred in late summer 1998, notably in Tuscany. This outbreak, attributed to a WNV-L1 strain, led to severe clinical cases among horses housed in close proximity to the Padule di Fucecchio [22]. Ten years after this initial report, in August 2008, WNV-L1 reappeared in Italy, in the Po River Delta area [23]. Unlike what occurred in Tuscany, this outbreak resulted in severe cases of neuroinvasive WNV among humans across multiple provinces in Emilia-Romagna and Veneto [24,25]. One possible explanation for the absence of earlier human cases could be the underestimation of WNV activity and the underreporting of West Nile disease in the country, particularly in the years leading up to the identification of the first human cases [26]. Between 2008 and 2011, WNV-L1 circulated constantly in Italy, impacting not only northern regions but also spreading southward, with cases reported in Sicily, Puglia, Calabria, Basilicata, and Sardinia [27,28,29]. In 2011, WNV-L1 first appeared in Sardinia, where it was responsible for a heightened occurrence of neurological cases in horses compared to the rest of Italy [28]. Moreover, during that timeframe, four human cases were documented, with three occurring in the province of Oristano and one in the province of Olbia-Tempio. Two of these cases resulted in fatalities [30]. In 2011, there was another significant development in the epidemiological evolution of WNV in Italy with the detection of WNV lineage 2 within the Italian territory. Since then, the circulation of WNV lineage 1 has predominantly been sporadic, with isolated detection reported in birds and mosquito pools in north-eastern regions (2012–2014, 2017), in Sardinia (2015–2016), and in Campania (2020) [31]. To date, both lineages are co-circulating.

Similarly to 2018, 2022 has been characterized by a high number of WND human cases. There were 588 confirmed cases, with 295 of them presenting neuroinvasive symptoms. In the same year, a total of eight cases of WNND were reported in Sardinia. Table 1 provides an overview of WN disease in humans in Italy over the past six years.

The guidelines of the PNA [5] typically advise collecting blood, urine, and/or cerebrospinal fluid samples to confirm WNV infection in humans. It is crucial to acknowledge that low-level or absent viremia at symptom onset and cross-reactivity of WNV antibodies with other flaviviruses may present challenges in swift diagnosis. However, in some cases, there is a contraindication to performing rickcentesis, as in the cases of patients on anticoagulant therapy or in very compromised clinical conditions; in other cases, a professional with the experience and skill necessary to carry out this invasive procedure is not immediately present in the treatment places.

In our case report, the clinical history of the patient highlights the rapid and severe course of WNV infection, progressing from initial symptoms of fever and tremors to neurological complications such as aphasia and hemiplegia in just 10 days, culminating in the diagnosis of encephalitis and death. While statistically only a small percentage of infected patients develop severe neurological symptoms associated with meningitis and/or encephalitis [10], it is crucial to understand that certain groups of individuals are at higher risk of severe complications. Specifically, patients over 60 years old and those with pre-existing medical conditions such as diabetes, hypertension, cardiovascular diseases, or immunosuppression, or as in this patient’s case, cancer, may be particularly susceptible to developing neurological complications and face the risk of death due to WNV infection.

In particular, patients undergoing immunosuppressive treatments such as chemotherapy or rituximab, as is often the case with those affected by lymphoma, are especially vulnerable. The profound immunosuppression in patients receiving rituximab leads to a rapid depletion of B cells (within 2–3 days post-infusion). It remains at low levels for up to 6 months and may take as long as 12 months to return to normal levels. This prolonged state of immunosuppression significantly increases the risk of severe complications. Experimental data suggest that both antibodies and B cells play a crucial role in preventing and controlling the early neurological dissemination of WNV, underscoring the importance of a robust immune response in effectively combating viral infections.

It is noteworthy that we were able to detect and sequence the WNV from nasopharyngeal swabs. Nasopharyngeal swabs have historically been considered the preferred specimen type for the detection of viruses, including SARS-CoV-2. To date, there is no evidence in the literature regarding the use of this type of sample for diagnosing WND in humans. Nonetheless, this approach in the context of arboviruses may offer a less invasive and more acceptable option for patients compared to traditional techniques, especially in situations where there is a contraindication to performing spinal tap or, like the one under study, where obtaining blood or cerebrospinal fluid samples is difficult or impractical.

The phylogenetic analyses of the detected WNV-L2 strain have essentially confirmed the previously reported observations [32]. In detail, two main groups were evidenced: the Southeastern Europe clade, which includes all sequences of strains that were initially introduced in Greece in 2010 and subsequently spread to the Balkans, and a second group, known as the Central Europe clade, which comprises isolated sequences from various countries, including Austria, Slovakia, Hungary, Germany, the Czech Republic, Serbia, and Italy (Figure 1). Within this clade, an Italian sub-clade has emerged, consisting of strains circulating in the country from 2012 to 2022 (Figure 2).

The strain isolated from this Sardinian woman is identified within a clade that comprise sequences exclusively isolated in Sardinia from 2015 to 2022 (Figure 2). The high degree of similarity with a sequence of another WNV strain detected in a goshawk (*Accipiter gentilis*) from the Sassari province highlights the potential for the zoonotic transmission of the virus from birds to humans through vectors, highlighting the importance of monitoring and controlling WNV outbreaks, particularly in regions where such circulation is observed. It also suggests a need for further investigation into the dynamics of WNV transmission within this specific territory to better understand and manage the risk to both wildlife and human populations.

## 6. Conclusions

In conclusion, the evolution of WNV from a minor pathogen to the most prevalent arbovirus globally underscores its escalating threat to public health in the context of globalization and climate change. Recent data from 2022 reveal a concerning trend, marked by a significant number of confirmed human WND cases, particularly those presenting with neuroinvasive symptoms. The case report presented highlights the swift progression and severity of WNV infection, especially in vulnerable individuals. In particular, since a patient who has received immunosuppressive therapy may have negative serological tests, including for WNV, PCR testing is essential for diagnosis. Thus, WNV disease should be considered in immunocompromised patients presenting with compatible symptoms. In light of this, this case report also suggests the utility of nasopharyngeal swabs as a less invasive option for sample collection, especially for patients for whom lumbar puncture is not feasible. Nevertheless, it is imperative to carefully assess the efficacy and reliability of this methodology in human patients and to compare it against traditional diagnostic methods. Further studies and research may be necessary to comprehensively evaluate the role of nasopharyngeal swabs in WNV diagnosis among human patients.

The phylogenetic analysis confirms previous findings, suggesting that the newly detected Sardinian strain likely originated from internal viral circulation within the region.

In summary, these findings underscore the dynamic nature of WNV transmission and emphasize the ongoing necessity for vigilant surveillance strategies, adaptability in response measures, and collaborative efforts. Additionally, it is crucial to monitor the emergence of new strains and the evolution of novel variants to effectively mitigate the public health impact of WNV.

## Figures and Tables

**Table 1:** WND epidemiological data in humans, with a breakdown of total cases, blood and organ donors, cases of West Nile Fever (WNF), cases of West Nile neuroinvasive disease (WNND), deaths, and cases recorded in Sardinia (https://www.epicentro.iss.it/westNile/bollettino, accessed on 19 September 2024).

Year	Total Cases	Blood/Organs Donor Cases	WNF	WNND	Deaths	Cases in Sardinia
2018	577	68	279	230	42	4
2019	56	7	24	25	5	0
2020	68	16	7	45	5	0
2021	55	15	5	35	0	0
2022	588	89	194	295	37	8
2023	332	72	70	190	27	3

## Data Availability

The original contributions presented in the study are included in the article, further inquiries can be directed to the corresponding author.

## Supplementary Materials

The following supporting information can be downloaded at: https://www.mdpi.com/article/10.3390/pathogens13111023/s1, Table S1: Dataset of 198 WNV-L2 reference sequences used for the phylogenetic analysis of WNV lineage 2 strains; Table S2: Primer pool 1 WNV-L2; Table S3: Primer pool 2 WNV-L2.
