# Supplementary material for: First Detection of West Nile Virus by Nasopharyngeal Swab, Followed by Phylogenetic Analysis
Source: Pathogens. 2024 Nov 20;13(11):1023. doi: 10.3390/pathogens13111023 (PMC11597865; doi:10.3390/pathogens13111023)
Supplement: Supplementary file 1 [file pathogens-13-01023-s001.zip › pathogens-3243828-supplementary.pdf]

# Supplementary Materials

**Table S1.** Dataset of 198 WNV-L2 reference sequences used for the phylogenetic analysis of WNV lineage 2 strains.

| GenBank | Country | Year |
|---|---|---|
| MH924836 | Germany | 2018 |
| KF179640 | Austria | 2008 |
| KM659876 | Austria | 2014 |
| KP109691 | Austria | 2014 |
| KP109692 | Austria | 2014 |
| MF984337 | Austria | 2015 |
| MF984338 | Austria | 2015 |
| MF984339 | Austria | 2015 |
| MF984340 | Austria | 2015 |
| MF984341 | Austria | 2015 |
| MF984342 | Austria | 2015 |
| MF984343 | Austria | 2015 |
| MF984344 | Austria | 2015 |
| MF984345 | Austria | 2015 |
| MF984346 | Austria | 2016 |
| MF984347 | Austria | 2016 |
| MF984348 | Austria | 2016 |
| MF984349 | Austria | 2016 |
| MF984350 | Austria | 2016 |
| MF984351 | Austria | 2016 |
| MF984352 | Austria | 2016 |
| KU206781 | Bulgaria:Sofia | 2015 |
| MT341472 | Bulgaria | 2018 |
| KM203860 | Cz Repubublic | 2013 |
| KM203861 | Cz Repubublic | 2013 |
| KM203862 | Cz Repubublic | 2013 |
| KM203863 | Cz Repubublic | 2013 |
| MT863560 | France | 2018 |
| MT863561 | France | 2018 |
| MH986055 | Germany | 2018 |
| MH986056 | Germany | 2018 |
| MW142223 | Germany | 2020 |
| MW142224 | Germany | 2020 |
| MW142226 | Germany | 2020 |
| MW142227 | Germany | 2020 |
| HQ537483 | Greece | 2010 |
| KF179639 | Greece | 2012 |
| KJ883343 | Greece | 2013 |
| KJ883344 | Greece | 2013 |
| KJ883348 | Greece | 2013 |
| KY594040 | Greece | 2010 |
| MN480792 | Greece | 2018 |
| MN480793 | Greece | 2018 |
| MN480794 | Greece | 2018 |
| MN480795 | Greece | 2018 |
| MN481589 | Greece | 2018 |
| MN481590 | Greece | 2018 |
| MN481591 | Greece | 2018 |
| MN481592 | Greece | 2012 |
| MN481593 | Greece | 2012 |

| GenBank | Country | Year |
|---|---|---|
| MN481594 | Greece | 2012 |
| MN481595 | Greece | 2010 |
| MN481596 | Greece | 2011 |
| MN481597 | Greece | 2012 |
| MN652880 | Greece | 2018 |
| MT341470 | Greece | 2019 |
| MT341471 | Greece | 2019 |
| DQ116961 | Hungary | 2004 |
| KC496015 | Hungary | 2010 |
| KT359349 | Hungary | 2014 |
| OK129329 | Hungary | 2016 |
| OK129330 | Hungary | 2016 |
| OK129331 | Hungary | 2015 |
| OK129332 | Hungary | 2015 |
| OK129333 | Hungary | 2017 |
| OK129334 | Hungary | 2017 |
| OK129335 | Hungary | 2017 |
| OK129336 | Hungary | 2017 |
| OK239658 | Hungary | 2018 |
| OK239659 | Hungary | 2018 |
| OK239660 | Hungary | 2018 |
| OK239661 | Hungary | 2018 |
| OK239662 | Hungary | 2018 |
| OK239663 | Hungary | 2018 |
| OK239664 | Hungary | 2018 |
| OK239665 | Hungary | 2018 |
| OK239666 | Hungary | 2018 |
| OK239668 | Hungary | 2018 |
| OK239669 | Hungary | 2017 |
| OK239670 | Hungary | 2018 |
| OK239671 | Hungary | 2018 |
| OK239672 | Hungary | 2018 |
| KP789953 | Italy:Lombardy Pavia | 2014 |
| KP789954 | Italy:Lombardy Cremona | 2014 |
| KP789955 | Italy:Veneto Verona | 2014 |
| KP789956 | Italy:Veneto Verona | 2014 |
| KP789957 | Italy:Lomabrdy Cremona | 2014 |
| KP789958 | Italy:Lombardy Pavia | 2014 |
| KP789959 | Italy:Lombardy Pavia | 2014 |
| KP789960 | Italy:Lombardy Mantova | 2013 |
| MN939558 | Italy:Veneto Rovigo | 2016 |
| KC496016 | Serbia | 2010 |
| MW751834 | Serbia | 2018 |
| MW751835 | Serbia | 2018 |
| MW751836 | Serbia | 2015 |
| MW751837 | Serbia | 2018 |
| MW751838 | Serbia | 2018 |
| MW751839 | Serbia | 2020 |
| MW751840 | Serbia | 2020 |
| MW751841 | Serbia | 2018 |

| GenBank | Country | Year |
|---|---|---|
| MW751842 | Serbia | 2018 |
| MW751843 | Serbia | 2018 |
| MW751844 | Serbia | 2018 |
| MW751845 | Serbia | 2018 |
| MW751846 | Serbia | 2018 |
| MH244510 | Slovakia | 2014 |
| MH244511 | Slovakia | 2013 |
| MH244512 | Slovakia | 2013 |
| MH244513 | Slovakia | 2013 |
| MW561633 | Slovakia | 2018 |
| OM037670 | Spain | 2017 |
| OM037671 | Spain | 2020 |
| OM037672 | Spain | 2020 |
| OM037673 | Spain | 2020 |
| MH939154 | Romania | 2014 |
| ON813230 | Italy:EmiliaRomagna | 2021 |
| ON813231 | Italy:EmiliaRomagna | 2021 |
| ON813232 | Italy:Sardinia | 2021 |
| ON813233 | Italy Piedmont | 2022 |
| OP850024 | Italy:EmiliaRomagna | 2022 |
| OP850025 | Italy Piedmont | 2022 |
| OP850026 | Italy:Friuli | 2022 |
| OP850027 | Italy:Lombardy | 2021 |
| OP850028 | Italy:EmiliaRomagna | 2021 |
| OP850029 | Italy:EmiliaRomagna | 2022 |
| OP734238 | Italy:EmiliaRomagna | 2022 |
| OP734239 | Italy:EmiliaRomagna | 2022 |
| OP734240 | Italy Piedmont | 2022 |
| OP734241 | Italy Piedmont | 2022 |
| OP734242 | Italy:EmiliaRomagna | 2022 |
| OP734243 | Italy:EmiliaRomagna | 2022 |
| OP734244 | Italy:Veneto | 2022 |
| OQ204314 | Italy:Tuscany | 2022 |
| OQ204315 | Italy:Sicily | 2022 |
| KF588365 | Italy:Veneto | 2013 |
| KF647248 | Italy:Veneto | 2013 |
| KF647249 | Italy:Veneto | 2013 |
| KF647250 | Italy:Veneto | 2013 |
| KF647251 | Italy:Veneto | 2013 |
| KF647252 | Italy:Veneto | 2013 |
| KF823805 | Italy:Veneto | 2013 |
| KF823806 | Italy:Lombardy | 2013 |
| KT207792 | Italy:Veneto | 2014 |
| KU573080 | Italy:EmiliaRomagna | 2013 |
| KU573082 | Italy:EmiliaRomagna | 2013 |
| MN939557 | Italy:Veneto | 2018 |
| MN939559 | Italy:Veneto | 2016 |
| MN939560 | Italy:Veneto | 2016 |
| MN939561 | Italy:Veneto | 2018 |
| MN939562 | Italy:Veneto | 2018 |

| GenBank | Country | Year |
|---|---|---|
| MN939563 | Italy:Veneto | 2018 |
| MN939564 | Italy:Veneto | 2016 |
| MW862073 | Italy:Sardinia | 2012 |
| MW862074 | Italy:Sardinia | 2013 |
| MW862075 | Italy:EmiliaRomagna | 2015 |
| MW862076 | Italy:Sardinia | 2015 |
| MW862077 | Italy:Sardinia | 2015 |
| MW862078 | Italy:Piedmont | 2015 |
| MW862079 | Italy:Piedmont | 2015 |
| MW862080 | Italy:Sardinia | 2015 |
| MW862081 | Italy:Sardinia | 2015 |
| MW862082 | Italy:Lombardy | 2015 |
| MW862083 | Italy:Sardinia | 2015 |
| MW862084 | Italy:Sardinia | 2015 |
| MW862085 | Italy:Sardinia | 2015 |
| MW862086 | Italy:Sardinia | 2016 |
| MW862087 | Italy:Sardinia | 2017 |
| MW862088 | Italy:Sardinia | 2017 |
| MW862089 | Italy:Lombardy | 2017 |
| MW862090 | Italy:Sardinia | 2018 |
| MW862091 | Italy:Sardinia | 2018 |
| MW862092 | Italy:Sardinia | 2018 |
| MW862093 | Italy:Sardinia | 2018 |
| MW862094 | Italy:Sardinia | 2019 |
| MW862095 | Italy:Sardinia | 2019 |
| MW862096 | Italy:Lombardy | 2018 |
| MW862097 | Italy:Sardinia | 2018 |
| MW862098 | Italy:Sardinia | 2018 |
| MW862099 | Italy:Sardinia | 2018 |
| MW862100 | Italy:Sardinia | 2018 |
| MW862101 | Italy:Sardinia | 2019 |
| MW862102 | Italy:Veneto | 2019 |
| MW862103 | Italy:Piedmont | 2019 |
| MW862104 | Italy:Piedmont | 2019 |
| MW862105 | Italy:Sardinia | 2019 |
| MW862106 | Italy:Piedmont | 2019 |
| MW862107 | Italy:Piedmont | 2019 |
| MW862108 | Italy:Piedmont | 2020 |
| ON032488 | Italy:Piedmont | 2020 |
| ON032489 | Italy:Piedmont | 2020 |
| ON032490 | Italy:Piedmont | 2021 |
| ON032491 | Italy:Lombardy | 2021 |
| ON032492 | Italy:EmiliaRomagna | 2021 |
| ON032493 | Italy:EmiliaRomagna | 2021 |
| ON032494 | Italy:Sardinia | 2021 |
| ON032495 | Italy:EmiliaRomagna | 2021 |
| ON032497 | Italy:EmiliaRomagna | 2021 |
| PP625328 | Italy:Sardinia | 2022 |

**Table S2.** Primer pool 1 WNV-L2.

| Primer Name | Sequence |
|---|---|
| WNV-L2_1_LEFT | GCCTGTGTGAGCTGACAAACTT |
| WNV-L2_1_RIGHT | TTCTTTTGTTTTGTGCTCCGGC |
| WNV-L2_3_LEFT | GTSYTRGCTGCTGGAAATGAYC |
| WNV-L2_3_RIGHT | CMACCCATGTAGCTCCAGAYAC |
| WNV-L2_5_LEFT | GTRGACAGRGGATGGGGAAAYG |
| WNV-L2_5_RIGHT | GRTTCCTCCAHGYGGTGCTT |
| WNV-L2_7_LEFT | GCDTTYAAATTCGCYGGGACTC |
| WNV-L2_7_RIGHT | GTGTATRGCTTTCCCYACYGAG |
| WNV-L2_9_LEFT | CGGTGYGGAAGTGGAGTGTTYA |
| WNV-L2_9_RIGHT | TRYTRTTCCATGCTCGGTTSR |
| WNV-L2_11_LEFT | CACHYTGTGGGGTGATGGAGTT |
| WNV-L2_11_RIGHT | CAGAAGGCCCAACTGAAAAGGA |
| WNV-L2_13_LEFT | CAGTCTTTCTGGTGGCTTCBTT |
| WNV-L2_13_RIGHT | YCCAGCKGCAAGTATCATBGGA |

| | |
|---|---|
| WNV-L2_15_LEFT | GGACRGCTGAYATYACYTGGGA |
| WNV-L2_15_RIGHT | RCTCATGAGAGCRGCTCCCTTR |
| WNV-L2_17_LEFT | RGACTATCCCACYGGAACRTCA |
| WNV-L2_17_RIGHT | TGGCACATGACATCAACGATYT |
| WNV-L2_19_LEFT | ACMGAGCCTGGAACACTGGVTA |
| WNV-L2_19_RIGHT | CCTCCATAGCAATACTCATCACCA |
| WNV-L2_21_LEFT | CWGTCTGGCTCGCTTACAAAGT |
| WNV-L2_21_RIGHT | ARGCTATTGTCTGAAGGGCRTC |
| WNV-L2_23_LEFT | YCAGCTYGCCGTGTTTTTGATY |
| WNV-L2_23_RIGHT | CYGCAGTCACAGTCACAGTCAG |
| WNV-L2_25_LEFT | ARRACTGTCAGAGAGGCYGGAA |
| WNV-L2_25_RIGHT | GCCTTTCCACTAACCACCGYAR |
| WNV-L2_27_LEFT | CACACTGCTCTGTGACATTGGA |
| WNV-L2_27_RIGHT | AGAATTGAGGAGAGGCTTCCCY |
| WNV-L2_29_LEFT | CTCCTTTCGGHCAACAACGRGT |
| WNV-L2_29_RIGHT | CYCCBAGCCACATGAACCADAT |
| WNV-L2_31_LEFT | CDAAGGTBCTTGARCTGCTDGR |
| WNV-L2_31_RIGHT | GGACCTTTGACATGGCATTBAGR |
| WNV-L2_33_LEFT | GCAGATGTGGCTGYTGCTTTAT |
| WNV-L2_33_RIGHT | YRTCTTCATACCTCCTCARDGA |
| WNV-L2_35_LEFT | GGAAGTTGAGTAGACGGTGCTG |
| WNV-L2_35_RIGHT | TCCCAGGTGTCAATATGCTGTT |

**Table S3.** Primer pool 2 WNV-L2.

| Primer Name | Sequence |
|---|---|
| WNV-L2_2_LEFT | ACAGCGATGAAGCATCTCTTGA |
| WNV-L2_2_RIGHT | GBCGDGTYTTDGTGCATCTYCC |
| WNV-L2_4_LEFT | ATNCTATTGCTCCTGGTRGCA |
| WNV-L2_4_RIGHT | TCCADCCAGTTGCTTTGGTKGW |
| WNV-L2_6_LEFT | CCTTCCTGGTYCACCGAGARTG |
| WNV-L2_6_RIGHT | KGAVGAAATGGGCACYTTRCAR |
| WNV-L2_8_LEFT | ACTCAGAGGAGCTCAACGACTC |
| WNV-L2_8_RIGHT | ATTYTTGCTAGGCCTTGTGGHG |
| WNV-L2_10_LEFT | YGCDCCAGARCTAGCTAACAAYA |
| WNV-L2_10_RIGHT | CCCTGGYCTCCTGTTRTGRTTGC |
| WNV-L2_12_LEFT | GAYGAAAAGACCCTCGTGCA |
| WNV-L2_12_RIGHT | CCATTTGRAAGAAAGCAGCTGCR |
| WNV-L2_14_LEFT | MRGTTGGAAGCYTCATCAARGARA |
| WNV-L2_14_RIGHT | TGAAAATTTCCATCRTCATCCARCC |
| WNV-L2_16_LEFT | ATHATGACTCGAGGTCTGCTYG |
| WNV-L2_16_RIGHT | MRCCATTDGGCATGATGACKCC |
| WNV-L2_18_LEFT | TKAGAGGACTTCCCATYCGGTA |
| WNV-L2_18_RIGHT | TTBCCCATTTTCACACTTGGAACR |
| WNV-L2_20_LEFT | TGATGGAAGAGTCATCCTGGGV |
| WNV-L2_20_RIGHT | GTGTTGGTTCGAGGTCCATCAA |
| WNV-L2_22_LEFT | TTKGACACGATGTATGTGGTKG |
| WNV-L2_22_RIGHT | CAYTCTTGGTCTTGTCCAGCCA |
| WNV-L2_24_LEFT | YTTTGTVGACGTTGGTGTGTCA |
| WNV-L2_24_RIGHT | TYGTTGCRTTCCACACTGARCT |
| WNV-L2_26_LEFT | RHGCCAGGAGAGAGGGAAAYRT |

| | |
|---|---|
| WNV-L2_26_RIGHT | CCARTCTTCCACCATCTCCARR |
| WNV-L2_28_LEFT | AAGAAAACVTGGAAGGGACCYC |
| WNV-L2_28_RIGHT | TBGTGGTCTCATTGAGGACGTR |
| WNV-L2_30_LEFT | ACYTGCATCTACAACATGATGGG |
| WNV-L2_30_RIGHT | GGBCTCATCACTTTCACGACYT |
| WNV-L2_32_LEFT | GGHGATGACTGYGTGGTDAA |
| WNV-L2_32_RIGHT | GRACCCAGTTVACAGGCACA |
| WNV-L2_34_LEFT | GCGCHACTTGGGCTGAAAAYAT |
| WNV-L2_34_RIGHT | MYCTTCCGAGACGGTTCTGA |
